# Severe Thrombocytopenia in a Pregnant Patient with Asymptomatic COVID-19 Infection: A Case Report

**DOI:** 10.7759/cureus.12990

**Published:** 2021-01-29

**Authors:** Maya L Moses, Nayla G Kazzi, Linden Lee

**Affiliations:** 1 Anesthesiology, McGovern Medical School, University of Texas Health Science Center, Houston, USA; 2 Obstetrics and Gynecology, McGovern Medical School, University of Texas Health Science Center, Houston, USA

**Keywords:** coronavirus, pregnancy, thrombocytopenia, covid-19, platelets, preeclampsia

## Abstract

Thrombocytopenia occurs in one-third of patients with coronavirus disease 2019 (COVID-19) infection and can indicate the severity of disease and may also increase the bleeding risk of performing invasive procedures. We present a pregnant patient with COVID-19 infection with the lowest platelet count described in the literature to date. The patient presented in labor at 38 weeks gestation with no other symptoms and was found to be positive on routine COVID-19 testing. The routine complete blood count upon admission was significant for a platelet count of 6 x 10^9^/L which was rechecked and resulted in a platelet count of 8 x 10^9^/L. The etiology of her thrombocytopenia was not clear prior to delivery as preeclampsia with severe features and lupus exacerbation were also possibilities that were considered. However, after delivery it became apparent that COVID-19 likely had a significant impact contributing to her severe thrombocytopenia. Her care was complicated by postpartum hemorrhage resulting in massive transfusion. This case highlights the importance of evaluating platelet count and coagulation status in COVID-19 patients, even if asymptomatic.

## Introduction

Coronavirus disease 2019 (COVID-19) caused by the virus severe acute respiratory syndrome coronavirus 2 (SARS-CoV-2) emerged initially in December 2019 and progressed into a global pandemic, infecting millions and killing hundreds of thousands worldwide [1]. The virus is transmitted primarily through the respiratory route, leading to highly variable clinical presentation and illness severity in those infected. While most of those infected are either asymptomatic or experience mild flu-like symptoms, severe systemic disease may occur somewhat unpredictably in others. Amongst this cohort with severe COVID-19, disease manifestations may include severe pneumonia, acute respiratory distress syndrome (ARDS), multiorgan failure, and disseminated intravascular coagulation (DIC) [1].

Thrombocytopenia may occur in patients with COVID-19 and is associated with significantly higher mortality. Studies have indicated that decreased platelet count is independently associated with both disease severity and risk of mortality in the intensive care unit [2]. The possible mechanisms of thrombocytopenia in COVID-19 patients are reduced platelet production, increased platelet destruction, or increased platelet consumption [3]. Thrombocytopenia occurs in one-third of patients with COVID-19 infection compared with 7%-12% of non-infected pregnant patients [4]. Depending on the severity and trend of thrombocytopenia, the risk of epidural hematoma following neuraxial anesthesia may outweigh the benefits of avoiding intravenous analgesia or general anesthesia. We present a case of an asymptomatic parturient with no history of thrombocytopenia who was admitted in labor, tested positive for COVID-19, and was found to have severe thrombocytopenia on routine complete blood count (CBC) collected at the time of admission. This article was previously presented as a poster at the 2020 American Society of Anesthesiologists Annual Meeting on October 3, 2020.

## Case presentation

A 36-year-old gravida 6 para 2 with a history of systemic lupus erythematous on prednisone 5 mg daily was admitted in spontaneous labor at 38 weeks gestation. She denied any respiratory symptoms upon admission but on mandated institutional rapid antigen screening tested positive for COVID-19. Admission vital signs included a temperature of 98.5°C, blood pressure of 134/97, respiratory rate of 18, and oxygen saturation of 93% on room air. The routine complete blood count collected upon admission was significant for a platelet count of 6 x 10^9^/L, which was rechecked and resulted in a platelet count of 8 x 10^9^/L. The patient had no prior history of thrombocytopenia and her platelet count two months prior was 245 x 10^9^/L. Preeclampsia with severe features was diagnosed upon admission secondary to persistent severe range blood pressures (defined as > 160/110 mmHg), an elevated urine protein/creatinine ratio (defined as > 0.3 mg/dL), and thrombocytopenia (defined as platelet count < 100 x 10^9^/L). Hemolysis, elevated liver enzymes, and low platelets (HELLP) syndrome was considered less likely given the patient’s liver enzymes (alanine aminotransferase 10 units/L, aspartate transaminase 20 units/L) and lactate dehydrogenase (117 units/L) values were within normal limits. Magnesium sulfate therapy was subsequently initiated for maternal seizure prophylaxis. Coagulation studies were within normal limits - protime was 13.0 seconds, partial thromboplastin time was 33.2 seconds, and international normalized ratio (INR) was 0.98. Fibrinogen was elevated to 700 mg/dL, above the normal pregnancy range of 200-600 mg/dl [5], which was suggestive of COVID-19 infection as inflammatory markers have been shown to be generally elevated. Lupus exacerbation was also considered as a possible etiology for her thrombocytopenia; however, the patient did not endorse any additional symptoms suggestive of a lupus exacerbation such as joint pain or cutaneous rashes.

Due to concerns of epidural hematoma with performing neuraxial anesthesia in a severely thrombocytopenic parturient, fentanyl patient-controlled analgesia (PCA) was ordered for labor analgesia. The patient had approximately 200 ml of vaginal bleeding during the first stage of labor. Placental abruption was suspected, however, fetal heart tones remained reassuring so a decision was made to proceed with her attempt at a vaginal delivery with close monitoring of her bleeding symptoms and clinical status. The patient was transfused a pooled unit of platelets which raised her platelet count to 81 x 10^9^/L. The patient eventually delivered vaginally but had significant vaginal bleeding postpartum. She received carboprost and misoprostol for uterine atony, but had persistent bleeding that was later thought to be from multiple cervical and vaginal lacerations, so the patient was taken to the operating room for an exam under anesthesia and repair of her lacerations. 

General anesthesia was induced and additional vascular access was obtained with a right radial arterial line and large bore peripheral intravenous lines. The massive transfusion protocol was initiated and the patient ultimately received a total of 12 units packed red blood cells, 12 units fresh frozen plasma, 4 units cryoprecipitate, and 4 pooled units of platelets. Thromboelastography revealed an increased thromboelastography activated clotting time (TEG-ACT), reaction time (R-time), and clot formation time (K-time), as well as a decreased a-angle, maximum amplitude, and clot stability (G-value). Multiple cervical and vaginal lacerations were repaired and a Bakri balloon was placed. The final blood loss was estimated at 7.5 L following resuscitation and coagulopathy correction. The patient was transferred to the intensive care unit, intubated and sedated. On postoperative day 1, she was extubated, the Bakri balloon was removed, and she was transferred out of the intensive care unit to the labor and delivery unit. However, she was readmitted to the ICU later that day due to an oxygen saturation of 85% on room air requiring oxygen supplementation via non-rebreather mask. She received remdesivir in addition to incentive spirometry and voluntary proning. She continued to improve clinically and was discharged home on postpartum day 6. At the time of discharge, the patient’s platelet count was 487 x 10^9^/L. 

## Discussion

Thrombocytopenia occurs in approximately one-third of patients infected with COVID-19 and can increase the bleeding risk of performing invasive procedures. While the risk of epidural hematoma with neuraxial anesthesia increases with worsening thrombocytopenia, obstetric anesthesiologists must carefully consider all factors including the respiratory status and severity of thrombocytopenia of pregnant patients infected with COVID-19 when making an anesthetic plan [6].

Our patient had multiple potential etiologies for her thrombocytopenia, including lupus, COVID-19 infection, and hypertensive disease of pregnancy. Hypertensive disease of pregnancy may cause severe thrombocytopenia in the case of HELLP syndrome; however, our patient did not exhibit signs of hemolysis or transaminitis. Lupus exacerbations may also cause thrombocytopenia, however, our patient’s disease had been otherwise well controlled and she did not demonstrate any other characteristic symptoms of a flare. In retrospect, COVID-19 infection was the most likely primary contributor to our patient’s thrombocytopenia. Her fibrinogen - an inflammatory marker characteristically increased in COVID-19 infection [7] - was elevated above normal levels during pregnancy. Her postpartum hemorrhage likely occurred as a result of consumptive coagulopathy from bleeding from multiple cervical and vaginal lacerations, although her thrombocytopenia certainly worsened the situation. Lastly, as the mechanism of thrombocytopenia in those with COVID-19 infection is not clear - and may include reduced platelet production, increased platelet destruction, or increased platelet consumption [3] - further investigation needs to be performed to determine how to best treat thrombocytopenic pregnant COVID-19 patients. 

This case represents the lowest platelet count of a pregnant patient with COVID-19 infection described in the literature to date and reinforces the recommendation of Bauer et al. [4] to obtain and review a recent platelet count in those with COVID-19 infection prior to performing a neuraxial procedure.

## Conclusions

Prior to the presentation, our patient had been asymptomatic and was incidentally found to have COVID-19 infection, severe thrombocytopenia, and preeclampsia with severe features. This case represents the lowest platelet count of a pregnant patient with COVID-19 infection described in the literature to date and reinforces the recommendation to obtain and review a recent platelet count in those with COVID-19 infection prior to performing a neuraxial procedure. It also demonstrates that COVID-19 infection may occur concurrently with hypertensive disease of pregnancy that may also cause thrombocytopenia, complicating the clinical picture. Once thrombocytopenia has been identified in a pregnant patient with COVID-19 infection, it may be worthwhile to obtain additional coagulation studies to further assess the feasibility of performing neuraxial procedures and to guide the correction of any coagulation abnormalities. 
